# Faster and more accurate pathogenic combination predictions with VarCoPP2.0

**DOI:** 10.1186/s12859-023-05291-3

**Published:** 2023-05-01

**Authors:** Nassim Versbraegen, Barbara Gravel, Charlotte Nachtegael, Alexandre Renaux, Emma Verkinderen, Ann Nowé, Tom Lenaerts, Sofia Papadimitriou

**Affiliations:** 1 Machine Learning Group, Université Libre de Bruxelles, 1050 Brussels, Belgium; 2 Interuniversity Institute of Bioinformatics in Brussels, Université Libre de Bruxelles-Vrije Universiteit Brussel, 1050 Brussels, Belgium; 3 Artificial Intelligence Laboratory, Vrije Universiteit Brussel, 1050 Brussels, Belgium

**Keywords:** Oligogenic diseases, Variant combinations, Pathogenicity predictor, Balanced random forest

## Abstract

**Background:**

The prediction of potentially pathogenic variant combinations in patients remains a key task in the field of medical genetics for the understanding and detection of oligogenic/multilocus diseases. Models tailored towards such cases can help shorten the gap of missing diagnoses and can aid researchers in dealing with the high complexity of the derived data. The predictor VarCoPP (Variant Combinations Pathogenicity Predictor) that was published in 2019 and identified potentially pathogenic variant combinations in gene pairs (bilocus variant combinations), was the first important step in this direction. Despite its usefulness and applicability, several issues still remained that hindered a better performance, such as its False Positive (FP) rate, the quality of its training set and its complex architecture.

**Results:**

We present VarCoPP2.0: the successor of VarCoPP that is a simplified, faster and more accurate predictive model identifying potentially pathogenic bilocus variant combinations. Results from cross-validation and on independent data sets reveal that VarCoPP2.0 has improved in terms of both sensitivity (95% in cross-validation and 98% during testing) and specificity (5% FP rate). At the same time, its running time shows a significant 150-fold decrease due to the selection of a simpler Balanced Random Forest model. Its positive training set now consists of variant combinations that are more confidently linked with evidence of pathogenicity, based on the confidence scores present in OLIDA, the Oligogenic Diseases Database (https://olida.ibsquare.be). The improvement of its performance is also attributed to a more careful selection of up-to-date features identified via an original wrapper method. We show that the combination of different variant and gene pair features together is important for predictions, highlighting the usefulness of integrating biological information at different levels.

**Conclusions:**

Through its improved performance and faster execution time, VarCoPP2.0 enables a more accurate analysis of larger data sets linked to oligogenic diseases. Users can access the ORVAL platform (https://orval.ibsquare.be) to apply VarCoPP2.0 on their data.

## Background

Identifying the causes of genetic diseases remains a key challenge in medical genetics, especially when more complex genetic patterns than the Mendelian ones are involved [1, 2]. Such patterns can include the co-presence of pathogenic variants in several genes leading to disease through their epistatic interactions (i.e. oligogenic diseases) [3–5]. Although the reporting on oligogenic cases has been rapidly advancing over the past years, the underlying epistatic mechanisms involved are still not well understood due to their complexity and the difficulty to replicate the obtained results [6]. Furthermore, the issue of high data dimensionality when investigating different gene and variant combinations as potential causative candidates makes their assessment even more challenging and time-consuming. In this regard, computational tools specifically tailored towards oligogenic data can offer an important aid by helping researchers focus on the most promising results and by promoting the detection of oligogenic patterns that would be difficult to uncover with the currently available methodologies.

The creation of the digenic disease database (DIDA) [7], which collected data on combinations of variants in two genes associated with a genetic disease (i.e. digenic or bilocus variant combinations), was an important step towards the collective understanding of oligogenic diseases and enabled the generation of novel predictive machine learning (ML) tools specialised in targeting the additive effect of single variants [8] or variant combinations directly [8–11]. Specifically, the Variant Combination Pathogenicity Predictor (VarCoPP) [11] was the first computational tool that used this data against variant combinations from the 1000 Genomes Project (1KGP) [12] and allowed for the classification of potentially disease-causing variant combinations in gene pairs based on a set of distinctive features at the variant, gene and gene pair levels. VarCoPP was integrated into the online platform ORVAL [13], which apart from predictions, incorporates explanatory annotations and predicted pathogenic gene networks for the exploration of potentially pathogenic oligogenic signatures. Since its publication, over 2000 unique users have accessed the platform, submitting over 20000 jobs, and research papers reporting promising pathogenic oligogenic combinations for genetic diseases using these methods have started to emerge [14–17]. Apart from VarCoPP, other ML methods have since appeared using DIDA [18, 19], which aim at a more general level to predict digenic gene pairs, rather than variant combinations.

Notwithstanding the usefulness and high accuracy of the first version of VarCoPP, still, several issues remained. VarCoPP presented a False Positive (FP) rate of 7% that limited its capacity to gene panels, as its application to exomes results in a combinatorial explosion of potential pathogenic combinations, requiring thus a significant amount of additional filtering. Another limitation was the complex architecture of its machine learning model, consisting of 500 different Random Forests (RFs). While this solution was proposed to solve class imbalance problems in machine learning methods [20], this also introduced significant computational requirements in terms of memory and computation time, especially for large gene panel or exome analyses for multiple patients. An alternative ML approach reducing this complexity would thus be welcome, while predictions should remain at least as good in terms of accuracy with the previous approach. Finally, the addition of more informative features depicting the relationship between the genes of a variant combination was important in order to better identify their epistatic relationships and improve the model’s sensitivity.

The creation of the oligogenic disease database (OLIDA) [21], which expands and improves DIDA by introducing confidence scores depicting the quality of evidence linking each variant combination to a disease, provided the foundations for the creation of a new and improved version of VarCoPP. We thus present here VarCoPP2.0, a new predictor that solves the aforementioned issues of the first version. This new version is trained on high-quality instances from OLIDA and uses an updated and diverse set of features through an improved feature selection procedure, as well as a simplified learning algorithm consisting of a single Balanced RF. VarCoPP2.0 demonstrates a significant improvement in performance both in terms of prediction and computational requirements, as it reduces the FP rate while increasing the sensitivity of the predictions, while also requiring significantly less time for computations. The model is available through the ORVAL platform.^1^

## Implementation

A summary table (Table 4) of the methodology in DOME recommendation style [22] is available in *Discussion & Conclusion*. Here we provide detailed information on the training data, features as well how they were selected for inclusion in the novel predictor. Model training with cross-validation, metrics, testing on an independent set and model interpretation are explained.

### Training data

To create the training set, we selected the bilocus variant combinations in OLIDAv1 (update January 2022), allowing for the presence of heterozygous compound variants (i.e. two different heterozygous variants) in the same gene. Based on the data present in OLIDA, we used variant combinations containing up to 2 variant alleles per gene. To ensure the creation of a high-quality data set, these combinations were further filtered to keep only those with an OLIDA FINALmeta score of $\ge$ 1, associated with the presence of at least weak evidence of oligogenicity [21], leading to 301 bilocus variant combinations constituting the positive training instances. For the neutral instances, 150,500 bilocus combinations were randomly selected from individuals of the 1000 genomes project (1KGP) [12], in order to be consistent with the 1:500 imbalance ratio that was used to train the original VarCoPP predictor. The 1KGP variants were filtered in order to obtain a set of variants similar to those in OLIDA. Variants with Minor Allele Frequency (MAF) > 3.5 % were removed, as well as intronic variants and synonymous variants that were not within 195 nucleotides from the exons boundaries. In order to remove any population bias, an equal number of individuals for each continent were selected, using the population data from the 1KGP project. For each 1KGP individual, a gene pair was selected at random, and for each gene within the gene pair, either one or two variants were also selected at random. Out of precaution, we excluded the 41 1KGP individuals who carried a variant combination found in the OLIDA database. These 14 OLIDA overlapping combinations had an OLIDA FINALmeta score of 0 and were thus also not included in the positive training set. The same approach was followed both for the selection of the training negative instances and for the validation set of 10,000 negative combinations. All data sets are available through GitHub: https://github.com/oligogenic/VarCoPP2.0.

### Annotation

The training instances were annotated with information at the variant, gene and gene pair levels, initially resulting in a set of 20 different features, as can be seen in Table 1. Features were chosen through a search in the scientific literature and were selected for our initial set based on their performance, their biological relevance, their maintenance and amount of missing values they would produce. Each training instance was represented as a feature vector where each element was linked to either a variant, a gene or the gene pair feature of the combination. Variant features are present four times - two variant features per gene to represent the two possible variant alleles inside the same gene, while gene features are present twice, one feature type per gene.

#### Variant level features

Each of the four variant alleles in a combination were annotated with the raw CADD score (CADD1, CADD2, CADD3 and CADD4, version 1.6 GRCh37) [23] as a measure of deleteriousness, as CADD is able to score many different types of variants including insertions and deletions (indels), it integrates different single variant prediction tools in a meta-prediction and is regularly updated. In the case of a heterozygous variant, where one of the alleles is wild-type, the CADD value was set to $-3$ as this falls outside its informative feature range.

#### Gene level features

The HIPred predictor [24], downloaded from https://github.com/HAShihab/HIPred, was used to annotate genes with a measure of haploinsufficiency (HIPred_A and HIPred_B, for gene A and B respectively).

The genes were also annotated with the dN/dS ratio, an evolutionary feature that quantifies the selection pressure of a gene by comparing the rate of non-synonymous mutations (dN) against the rate of synonymous mutations (dS) [25]. dN and dS values were downloaded for each gene in human and 9 different organisms from Ensembl Biomart version 99 and the final value was computed as the mean of the dN/dS ratios over all the different organisms (dN_dS_A and dN_dS_B).

Finally, the Inheritance Specific Pathogenicity Predictor (ISPP) [26] was used to annotate genes with three measures of pathogenicity under specific inheritance patterns, autosomal dominant (AD), autosomal recessive (AR) and X-linked (XL). Each gene was annotated with each of the three scores (ISPP_AD_A, ISPP_AD_B, ISPP_AR_A, ISPP_AR_B, ISPP_XL_A, ISPP_XL_B). Since genes that are not located on the X chromosome can not be attributed an X-linked score, their ISPP_XL score was set to a negative value of $-0.5$ as this falls outside of the informative feature range (0–1).

#### Gene pair features

Gene pairs were annotated with the biological distance (Biol_Dist) [27], a feature which was already incorporated in the first version of VarCoPP and is a measure of distance between two genes in the STRING protein-protein interaction network.

Coexpression values (Coexp) for the genes comprising a gene pair were obtained from the COeXPRESsed gene DataBase (COXPRESdb), version 8.0 [28].

The gene ontology (GO) similarity between two genes was computed for each subgraph of the gene ontology (Biological Process, Molecular Function and Cellular component) independently, thus leading to three different scores respectively (BP_Sim, MP_Sim and CC_Sim). The software designed to compute the Human Phenotype Ontology (HPO) similarity^2^ was adapted for GO terms and the similarity was calculated using the SimGIC score with the Best-Match Average (BMA) method, as it has been shown to provide more accurate results [29].

Finally, the gene pairs were annotated with a distance measure obtained from an in-house developed heterogeneous oligogenic knowledge graph (KG_Distance), which aggregates information about different types of relationships between genes. Links between gene pairs and disease were obtained from OLIDA [21], protein-protein interactions (PPI) were collected from the Mentha database [30], coexpression between genes was obtained from post-processed Gtex data collected in the TCSBN database [31], sequence similarity data between genes was downloaded from STRING [32], pathway information was obtained from Reactome [33], gene ontology terms and their linked genes were collected from the Gene ontology [34, 35], protein family information was collected from the InterPro database [36] and protein complexes information was gathered from CORUM [37]. The distance between two genes is computed as the length of the shortest path between the genes in the graph using the Dijkstra algorithm[38] and then divided by the number of different types of nodes in the graph that are part of the path, in order to take into account the heterogeneity of the graph: e.g. if two genes are part of the same pathway (i.e. they are both connected to the same pathway node in the graph), the length of the path between these genes is 2 and the knowledge graph distance value is 1 since there are two types of nodes (Gene and Pathway nodes) in the path. For two genes that are directly interacting through PPI, the knowledge graph distance is also 1, since the length of the shortest path between these genes is 1 and there is only one type of node involved.

#### Imputation and variant order

In order to obtain consistent annotations across all variant combinations, the genes inside each combination were ordered by the Residual Variation Intolerance Score (RVIS), a measure of gene intolerance to mutation [39], with the more intolerant gene (i.e. the gene with the lower RVIS) being assigned first, as gene *A*. The alleles of heterozygous variants inside a gene were ordered using the CADD score, with the most deleterious variant (i.e. the variant with the higher CADD) being first.

For each type of feature, missing values were imputed with the average of the median of the feature value obtained from each training set (i.e. if the HIPred value is missing for a gene, it is replaced by the mean of the median HIPred value for the positive data set and the median HIPred value for the negative data set). When the value of a feature was missing because it could not be calculated (e.g. ISPP_XL score for a gene that is not located on the X chromosome), a negative value was imputed.

### Feature selection

A feature selection procedure, based on a heuristic optimisation approach, was applied to identify the most relevant features from the total set of 20 leading to the selection of the 15 most relevant features (see Table 1). The feature selection among the set of the 20 potential features was translated into a heuristic optimisation problem using a wrapper approach [see e.g. 40]. The search was formulated as a relaxed version of the full VarCoPP classification problem by only considering the 301 OLIDA (positive) and 301 random 1KGP (negative) instances to train one Random Forest (RF) with 100 trees. Performance, assessed by the mean F1 score of a 5-fold cross validation, was the objective function to maximise and the search space included all possible subsets of features. The step function consisted of inverting the state of a feature (i.e. including it in the set of features used for training if it was excluded and vice versa). At each iteration, 10 random neighbors (i.e. one step removed from the current set) were generated and evaluated. To avoid redundant computation, memoization was used to store and retrieve the result of the performance computation for each considered set. The search was restarted 10,000 times and each time a different random starting point was selected in the search to avoid local maxima. Each search continued until the performance remained stable after 100 successive search steps.

The features that were selected in the final model are represented in bold in Table 1.

**Table 1:** Set of features that were considered during the creation of VarCoPP2.0

Feature	Feature abbreviation	Feature description	PMID	Version
CADD raw score	**CADD1**	CADD score variant 1 of gene A	24487276	CADDv1.6
	**CADD2**	CADD score variant 2 of gene A		
	**CADD3**	CADD score variant 1 of gene B		
	**CADD4**	CADD score variant 2 of gene B		
Haploinsufficiency prediction	**HIPred**_**A**	HIPred of gene A	28137713	N.A.
	**HIPred**_**B**	HIPred of gene B		
	**ISPP**_**AD**_**A**	ISPP prediction for AD mode of inheritance for gene A	27354691	N.A.
	**ISPP**_**AD**_**B**	ISPP prediction for AD mode of inheritance for gene B		
Inheritance mode specific	**ISPP**_**AR**_**A**	ISPP prediction for AR mode of inheritance for gene A		
pathogenicity prediction	**ISPP**_**AR**_**B**	ISPP prediction for AR mode of inheritance for gene B		
	**ISPP**_**XL**_**A**	ISPP prediction for XL mode of inheritance for gene B		
	ISPP_XL_B	ISPP prediction for XL mode of inheritance for gene B		
Selection Pressure (dN/dS)	**dN**_**dS**_**A**	Selection pressure for gene A	26896847	v99
	dN_dS_B	Selection pressure for gene B		
Biological distance	**Biol**_**Dist**	Biological distance between gene A and gene B	24694260	v12.2015
Coexpression	Coexp	Coexpression value between gene A and gene B	30462320	COXPRESdbv8.0
	**BP**_**sim**	Biological Process similarity	10802651	GO release 2021-12-15
Gene ontology similarity	MF_sim	Molecular Function similarity	18460186	
	CC_sim	Cellular Component similarity		
Knowledge Graph distance	**KG**_**distance**	Distance between gene A and gene B in		N.A.
		an in-house developed knowledge graph		

Abbreviations as used in the data files for respective features can be found in the Feature abbreviation column. The PMID column provides the PMID of the relevant citation for each feature. The version of each feature used in this work is listed in the Version column. Features that are selected in the final model are marked in bold

### Model training and cross-validation

The original version of VarCoPP was an ensemble model consisting of 500 RFs. An important objective of this work was to evaluate whether less computationally demanding models would be able to achieve similar performance. To that end, different model structures were examined in order to create the final model: a single RF [41], a single Balanced RF [42] and different ensembles of RFs (similarly to the initial version of VarCoPP), where the number of trees, tree depth and class imbalance ratios varied. For the Balanced RF the imbalance ratio remained stable (1:500) as all the training data was available. The empirical evaluation of these model structures resulted in choosing a Balanced RF containing 400 Decision trees of depth 10 [42] using the imblearn package [43], using as training data sets the 301 OLIDA and the 150,500 1KGP variant combinations. In this type of model, for each tree, a bootstrap sample is drawn from the minority class and the same number of majority class instances are drawn randomly with replacement. As such, this procedure down-samples the majority class (i.e. the 1KGP combinations).

#### Cross-validation

The various model structures that were considered, were evaluated by comparing the Average Precision Score (APS) for each parameter variation in a 5-fold cross validation. The final model was evaluated using a stratified Leave-One-Group-Out (LOGO) cross-validation on the training data, with the gene pairs being the stratification groups. At each fold, dictated by a particular gene pair, the instances in the training data belonging to that pair were left out from the training data and were used as a test set. Different evaluation metrics were then applied to the aggregate predictions of all folds.

#### Validation

To validate the performance of the predictor we used two independent data sets. The positive data set comprised 53 bilocus combinations that were added during a subsequent update of OLIDA and were assigned a FINALmeta score $\ge$ 1 [see 21]. These combinations are available through GitHub (https://github.com/oligogenic/VarCoPP2.0). The neutral independent data set contained 10,000 1KGP bilocus combinations, not included in the training set, which were selected from individuals of the 1KGP in the same way as the training set. Instances of the independent sets were predicted using the model trained on the entire training data and using the probability threshold of 0.5.

### Evaluation metrics

Considering the context of pathogenic bilocus variant combination prediction, one is faced with a huge data imbalance; the vast majority of bilocus variant combinations one can generate are not associated with a disorder, while a small minority are. We thus adopted evaluation metrics suited to evaluate the performance of the predictor in a hugely imbalanced setting (1:500 ratio, with positive instances forming the minority class). Precision, recall, specificity, F1-score and geometric mean were computed per-class and were aggregated according to the number of class instances (i.e. weighted by support):

- Precision: $$tp / (tp + fp)$$
- Recall: $$tp / (tp + fn)$$
- Specificity: $$tn / (tn + fp)$$
- f1: $$2 * (precision * recall) / (precision + recall)$$
- Geometric mean: $$\sqrt{Recall_- \times Recall_+}$$

Where $Recall_-$ indicates the recall for the negative class and $Recall_+$ the recall for the positive class.

### Model interpretation

The model interpretation was performed on the final model using both global and local model interpretation methods. As a global interpretation method, feature importance of the trained model was examined using the Gini importance, where the reduction in Gini impurity is calculated for each tree composing the RF. Local model interpretations (i.e. on specific samples), were carried out using *treeinterpreter*,^3^ which separates each prediction into a fixed bias and a contribution specific to the sample being examined, for each feature. The results of these analyses are available in the *Feature importance* section.

### Confidence zones

In the first version of VarCoPP, confidence zones were defined to provide users with a sense of how many FPs they could expect; a 95% zone and a 99% zone, in which a user could expect 5% FPs and 1% FPs respectively. To do so, a large number of neutral combinations, distinct from those used for training the model, were tested and thresholds on the prediction probabilities were determined based on a desired (5% and 1%) FP density. For VarCoPP2.0, we selected 10,000 neutral combinations distinct from the training set, following the same selection principles as for the neutral training set (see “Training data” section), and defined two confidence zones; a 99% zone and a 99.9% zone, in which users can expect 1% FPs and 0.1% FPs respectively.

The code and data for these experiments are available at https://github.com/oligogenic/VarCoPP2.0.

## Results

### Balanced random forests outperform more complex model structures

In this work we present VarCoPP2.0, an improved version of VarCoPP [11] in terms of performance and computational time, which classifies bilocus variant combinations into disease-causing or neutral. The model is trained based on 301 high-quality curated bilocus variant combinations from OLIDA linked to oligogenic diseases as a positive set [21] and 150,500 bilocus variant combinations from the 1KGP as a negative set [12] (Both sets can be found online through GitHub: https://github.com/oligogenic/VarCoPP2.0).

The first version of VarCoPP was an ensemble model consisting of 500 RFs, a structure shown to be relevant for dealing with a severe class imbalance [20]. For VarCoPP 2.0, various model structures were evaluated in order to choose the one with the optimal performance. We considered a single RF [41], a single Balanced RF [42] and various ensembles of RFs, where the number of trees, the tree depth and the class imbalance ratio also varied. For the Balanced RF, where the majority class is randomly down-sampled in each tree to match the minority class, the imbalance ratio remained stable (1:500) as the RF used as input all available training data. We evaluated the performance of these models with the use of confusion matrices and APS in a cross validation setting (see Section *Cross-validation*).

**Fig. 1:**
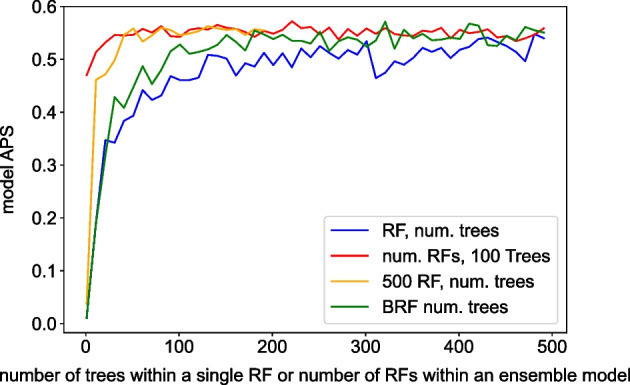
ROC-AUC for various model structure variations. The blue line represents the evolution of APS when varying the number of decision trees in one single RF. The input for the RF was the entire set of positive instances and a balanced random sample (1:1 ratio) of negative instances. The green line represents the evolution of APS for a single balanced RF, where the number of decision trees composing the balanced RF varies. The input for each Balanced RF was the same full training data set (with it’s 1:500 imbalance ratio). The orange line represents the evolution of APS when varying the number of decision trees present in each of the 500 RFs in an ensemble RF model, similarly to the first version of VarCoPP. The input for each RF was the entire set of positive instances and an equal amount of negative instances, specific to each RF. The red line represents the evolution of APS for different numbers of RFs in an ensemble RF model. Each RF consisted of 100 decision trees and its input was the entire set of positive instances and an equal amount of negative instances, unique for each RF

Though the ensembles of RFs perform relatively well with a moderate number of forests or trees, the BRF matches their performance in terms of APS given sufficient trees composing it, i.e. 400 trees (Fig. 1). After this point, its performance reaches a plateau. On the other hand, the single RF consistently under-performs compared to the other models. As the BRF is considerably less complex than the two ensemble models, while showing at the same time similar or better performance with sufficient number of trees, we opted for a BRF with 400 trees as the final model for VarCoPP2.0,

### VarCoPP2.0 shows increased sensitivity and a lower FP rate

The performance of the Balanced RF model of VarCoPP2.0 was evaluated in both cross-validation and independent validation settings, with the measured metrics for both settings being shown in Table 2.

**Table 2:** Classification report for the stratified LOGO cross validation and the independent validation set per class

		pre	rec	spe	f1	geo	Support
LOGO	Neutral	1.00	0.95	0.93	0.98	0.94	150,500
	Disease-causing	0.04	0.93	0.95	0.07	0.94	301
	avg/total	1.00	0.95	0.93	0.97	0.94	150,801
VAL	Neutral	1.00	0.95	0.98	0.98	0.97	10,000
	Disease-causing	0.10	0.98	0.95	0.19	0.97	53
	avg/total	1.00	0.95	0.98	0.97	0.97	10,053

The *LOGO* rows indicate the results obtained during the stratified LOGO cross validation, while the *VAL* rows indicate the results obtained for the independent validation set. The bottom row presents a weighted (relative to the number of positive and negative instances) average of the metrics. Pre:precision, rec:recall, spe:specificity, geo:geometric mean, support: number of class instances (see “Evaluation Metrics” section for the definition of these metrics)

**Fig. 2:**
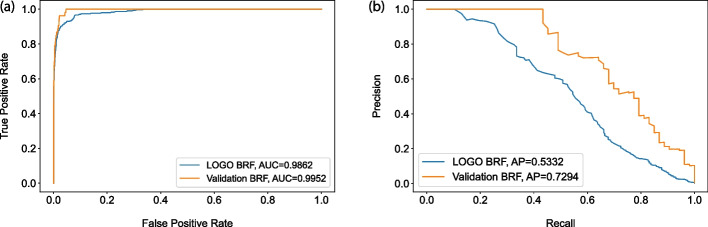
ROC- and PR-curve for LOGO cross-validation and independent validation: **a** ROC-curve based on Balanced RF prediction probabilities in both stratified LOGO cross validation (Blue) and validation set (Orange) settings. **b** PR-curve based on Balanced RF prediction probabilities in both stratified LOGO cross validation (Blue) and validation set (Orange) settings

The cross-validation results (LOGO) indicate that VarCoPP2.0 classifies bilocus variant combinations with high accuracy, achieving a TP rate of 0.95 and a FP rate of 0.05. Additional performance metrics suited for the class imbalance shown in Table 2, as well as the computed ROC-AUC of 0.986 and the PR-AUC of 0.5 (Fig. 2) demonstrate that the predictor performs very well in this cross-validation setting.

In order to confirm the good performance of VarCoPP2.0 on novel data, an independent validation data set containing both new positive and negative instances was created (see *Methods*), on which the model, trained on the complete training data set, was applied using the default 0.5 prediction threshold. As can be observed in Table 2 and Fig. 2, VarCoPP2.0 also shows great sensitivity on that set, being able to classify 52 out of 53 OLIDA combinations as disease-causing, and mis-predicting only $5\%$ of the neutral combinations, similarly to its performance during the cross-validation. The sensitivity of VarCoPP in this setting is particularly important, as in medical genetics it is crucial to be able to detect the pathogenic variants if these are present, and indeed causative, in the patient. A ROC-AUC of 0.995 and a PR-AUC of 0.7294 is obtained in the independent validation setting (Fig. 2)

**Fig. 3:**
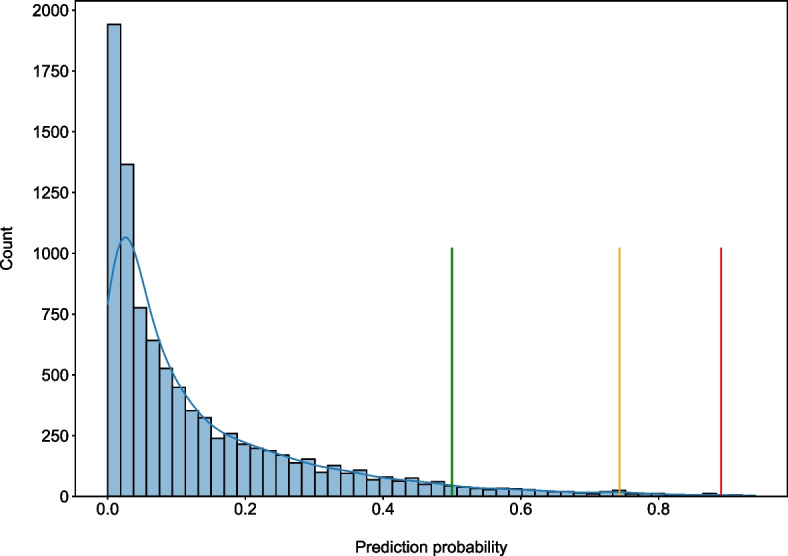
Density plot of the prediction probabilities for the 10,000 neutral 1KGP variant combinations used as a negative validation set. The X-axis represents the prediction probabilities, while the Y-axis shows the number of instances that were assigned the corresponding probability score. The plot visually presents the Disease-causing (green vertical line), $99\%$ (orange vertical line) and $99.9\%$ (red vertical line) confidence zones’ thresholds, indicating that 1% or less of all samples are to the right of the green line (i.e. were assigned a higher probability) and similarly 0.1% or less of all samples are to the right of the red line

Similarly to the first version of VarCoPP, we defined confidence zones for the predictions as a way to link the expected number of FPs with particular prediction thresholds, based on the performance of the predictor on the neutral independent set. Given the fact that VarCoPP2.0 predicts less FPs than the first version, three confidence zones were defined: Disease-causing - when a prediction is predicted as positive based on the classification threshold 0.5, the 99% confidence zone—where at most 1% of predictions could be FPs, for which the classification threshold is 0.743, and the 99.9% confidence zone—where 0.1% of predictions could be FPs, for which the classification threshold is 0.891 (Fig. 3). As a result, medical researchers can decide the threshold that can be used to assess and/or further filter the results derived from VarCoPP2.0 based on their use-case and a strictness level; the minimal optimal probability threshold that is set by default and is based on a minimisation of FPs and a maximision of TPs, the 99% zone threshold or the 99.9% zone threshold.

Application of these confidence zones in the independent positive set of OLIDA shows that 30/53 (56%) of the disease-causing combinations fell directly into the 99.9% zone, while 45/53 (85%) fell also into the 99% confidence zone, highlighting the usability of these confidence zones and indicating that VarCoPP2.0 provides predictions of high confidence for known variant combinations linked to disease.

### VarCoPP2.0 displays significant computational time improvements

The validation set of both positive and negative instances was used to compare the performance of VarCoPP2.0 to the original VarCoPP model that is trained on the DIDA data and is composed of 500 RFs (see Table 3). In order to examine the impact of the OLIDA data set on the predictions, we further compared VarCoPP2.0 to the original VarCoPP model trained with the OLIDA data as well (VarCoPP OLIDA). The results of the comparison (Table 3) demonstrate that although the inclusion of the higher quality data of OLIDA does not significantly increase the performance, even though a noticeable decrease in FPs was observed, it is the choice and addition of new gene and gene pair features and the new model structure of VarCoPP2.0 that enable us to give more confident predictions. VarCoPP2.0 is superior to the initial version of VarCoPP in terms of FPs, by reducing the number of FP validation instances to 452 compared to 687, as well as in terms of sensitivity, being able to correctly classify 52 out of 53 novel OLIDA combinations of the validation set as pathogenic, compared to 44 for the original VarCoPP.

**Table 3:** Confusion matrices comparing VarCoPP2.0 with the first version of VarCoPP trained on DIDA (original version) and on OLIDA instances (VarCoPP OLIDA)

		Output Class					
		VarCoPP		VarCoPP OLIDA		VarCoPP2.0	
		$+$	−	$+$	−	$+$	−
**Target Class**	$+$	44	9	43	10	52	1
	−	687	9313	654	9346	452	9548

$+$ indicates the positive, disease-causing, class and − the negative, neutral, class

**Fig. 4:**
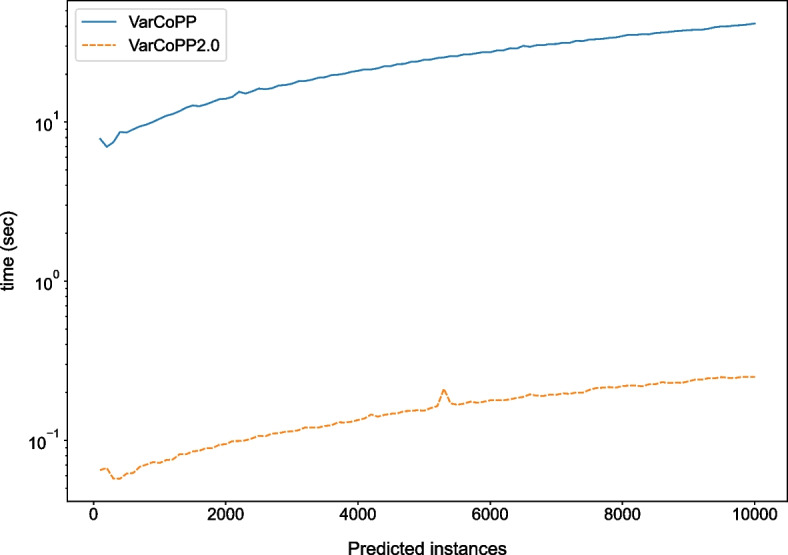
Comparison between VarCoPP and VarCoPP2.0 in terms of execution time needed to classify a certain number of variant-combinations (x-axis), shown with logarithmic y-scale

Furthermore, a particularly important improvement of the new model is its simplicity without sacrificing performance, as the complexity has decreased from 500 different RFs to one balanced RF model with 400 decision trees. This change translates into huge differences in the time required for predictions, especially when a large number of bilocus combinations need to be tested (Fig. 4). VarCoPP2.0 creates a dramatic reduction in the required computational time as it predicts samples around 150 times faster on average compared to the first version.

### Predictions now rely more heavily on gene pair information

**Fig. 5:**
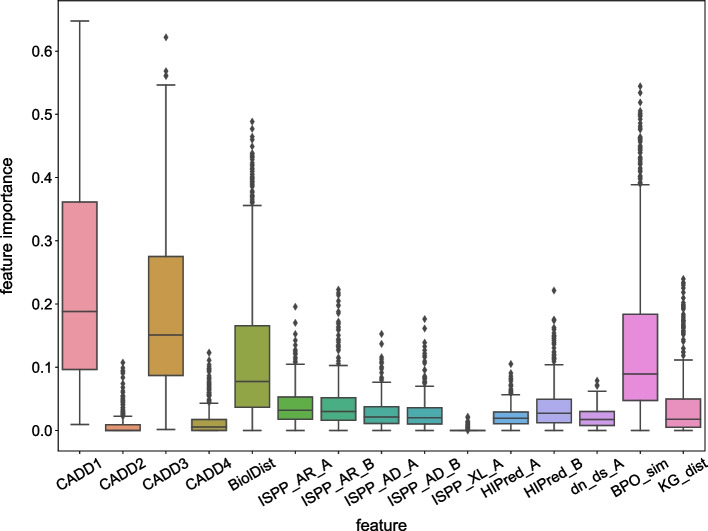
Boxplot of the Gini importance per feature in the Balanced RF trained on the entire set of training data. A higher Gini importance value indicates a higher contribution for the prediction (regardless of whether the prediction is positive or negative)

In order to further examine the contribution of the chosen features for predictions and the potential existence of any bias, the individual feature importance of the trained model was examined using the Gini importance measure. A Gini importance boxplot is presented for each feature over all trees of the balanced RF in Fig. 5, providing a global model interpretation. This interpretation reveals that CADD1 (i.e. the CADD score of the first variant allele of gene A), CADD3 (i.e. the CADD score of the first variant allele of gene B), Biol_Dist and BP_similarity are the most important features for prediction.

Compared to the original version of VarCoPP, we observed that although variant-related features, such as the CADD1 and CADD3, remain two of the most important drivers for predictions, all gene pair features in VarCoPP2.0 (Biol_Dist, BP_similarity and KG_dist) emerge as the second most important predictive feature group. This demonstrates that VarCoPP2.0, compared to the original VarCoPP, utilises more the biological relatedness information between genes and that, these features, as shown above, contribute to the better performance of the model. An important difference is also that the original VarCoPP model was further stratified using the degrees of separation between genes (i.e. the number of proteins connecting the protein products of the genes in a pair inside a protein-protein network), a decision that had made VarCoPP less sensitive to gene-pair related information differences between the neutral and the disease-causing set.

**Fig. 6:**
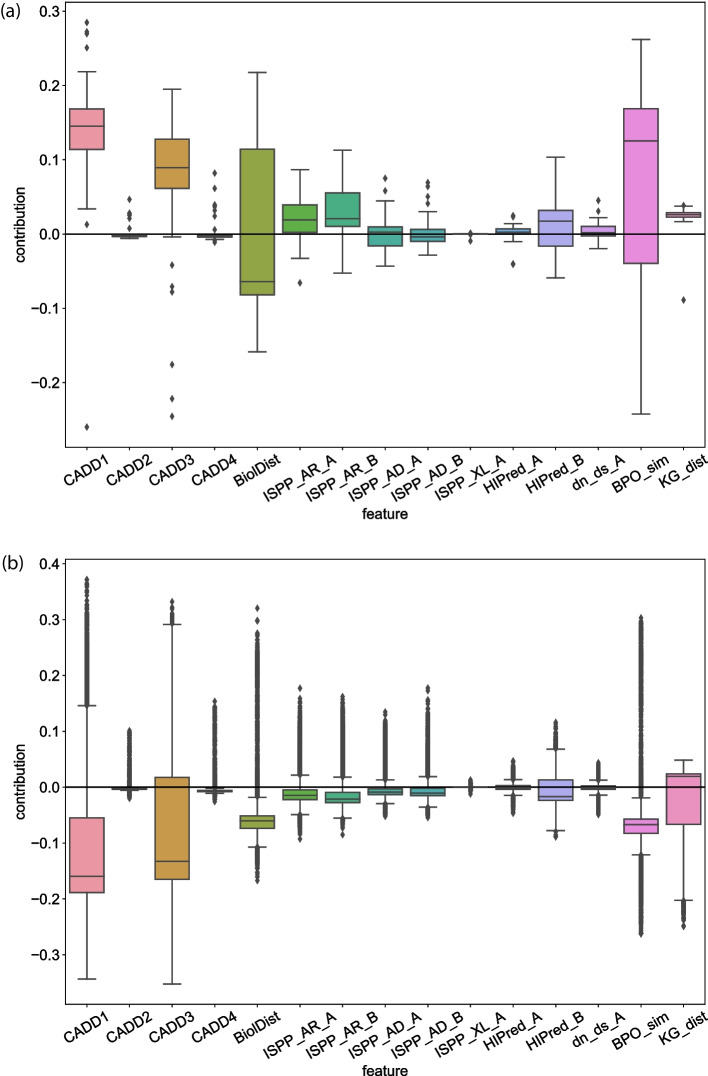
Boxplot of the feature contributions for either the disease-causing or the neutral class, among all positive instances (**a**) or all negative instances (**b**) in the validation set inferred using *treeinterpreter*. A feature contribution value above 0 indicates a vote for a positive prediction (i.e. towards the disease-causing class), while a value below 0 indicates a vote for negative prediction (i.e. towards the neutral class). The more the feature contribution value deviates from 0, the stronger the vote is for either class

Moreover, we examined local model interpretations inside the model (i.e. on specific instances), using *treeinterpreter*.^4^ This package separates each prediction into a fixed bias value and contribution values linked to each of the used features, specific to a particular instance being examined. We observed that these local model interpretations are consistent with the global model interpretation for both the positive and the negative data set, in the sense that CADD1, CADD3, Biol_Dist and BP_similarity seem to drive the predictions (or, vote) the most in the negative direction (i.e. towards the neutral class) for the negative instances and towards the positive direction (i.e. towards the disease-causing class) for the positive set (Fig. 6). Noteworthy is that variation seems relatively high in both BP_similarity and Biol_Dist contributions for the positive instances.

## Discussion and conclusion

The development of the VarCoPP2.0 predictor is an important next step in the field of combinatorial variant pathogenicity predictions and offers significant improvements in terms of both classification performance and computational time, compared to the first VarCoPP predictor published in 2019. Such improvements are essential in order to obtain predictions of better quality, especially when taking into account the fact that this framework is already being used by medical researchers to investigate oligogenic signatures using sequencing data of their patients.

The main innovations of the VarCoPP2.0 model include the use of a higher-quality positive training data set, the integration of better calibrated biological features, the use of a more optimal feature selection method and the selection of a simpler model structure. The creation of an improved training set was made possible with the publication of the OLIDA database, which assigns confidence scores to variant combinations involved in oligogenic diseases reported in literature. The combinations used to train VarCoPP2.0 could therefore be selected based on these confidence scores, allowing for only those instances that are linked to evidence for oligogenicity to be included.

VarCoPP2.0 accurately predicts 95% of the OLIDA combinations as disease-causing in cross-validation, while it correctly predicted 52 of the 53 of the independent disease-causing combinations used as a test set. This improved sensitivity of the predictor is particularly important for clinical applications, where it is crucial that all pathogenic instances are detected. Moreover, the model presents a 5% FP rate, which is lower than the 7% of the initial VarCoPP predictor, indicating that less combinations now need to be further filtered by the users. In case post-prediction filtering is still needed, we provide 99% and 99.9% confidence zones that allow for the filtering of the predictions based on the number of expected FPs.

The new feature set was obtained by searching the literature for recent and up-to-date features representing different biological levels, with a special focus on enriching the model with more gene-pair level features compared to the first version of VarCoPP, in order to better capture the gene-gene synergistic relationship inside a variant combination. The observation that now both variant and gene-pair level features are among the 5 most important predictive features indicates that a good integration of characteristics from different biological levels is particularly important for improved performance. The new model structure, a balanced RF with 400 trees, represents a substantial reduction of complexity compared to the first version of VarCoPP, and removes the necessity of using two separate scores to classify samples, thus rendering the thresholding for specific purposes (e.g. specific gene panel requirements) more straightforward. Furthermore, this simpler model structure drastically reduces the computational time required for predictions, which is especially important when a large number of variant combinations needs to be analysed, such as in the context of whole-exome or whole-genome sequencing analyses.

As a result of the constructed feature representation of the model and the limited amount of higher-order oligogenic cases involving more genes with confident pathogenic evidence that are currently present in OLIDA [44], VarCoPP2.0 remains constrained to the prediction of bilocus variant combinations. However, the ORVAL platform [13], where VarCoPP2.0 is integrated, allows for the exploration of oligogenic networks that may involve more than two genes, by linking gene pairs containing predicted pathogenic variant combinations. As such, ORVAL allows for the exploration of potential higher-order oligogenic networks through the predictions of VarCoPP2.0.

With the advent of more data on oligogenic cases we aspire to continue improving the performance and the applicability of the predictor by including, for example, non-coding variants that are currently not well represented in the training sets and modifier variants of higher frequency, as at the moment relatively rare variants of up to 3.5% MAF were included during training based on the information available in OLIDA.

**Table 4:** DOME Table consisting of essential information to assess the machine learning approach [22]

VarCoPP2.0	Version	2.0
Data	Provenance	OLIDA [21] and 1000 genomes Project [12]. 1:500 ratio
	Dataset splits	301 positive instances, 150,500 negative instances for training data. 53 positive and 10000 negative instances for validation set. Training with stratified LOGO cross-validation
	Redundancy between data splits	No overlap
	Availability of data	Yes: olida.ibsquare.be (new curated data will be added) and www.internationalgenome.org
Optimization	Algorithm	Balanced Random Forest
	Meta-predictions	Yes: CADD features and ISPP features stem from a predictive model
	Data encoding	Global features
	Parameters	400 decision trees within RF
	Features	15 features, obtained through wrapper approach on training data only, using mean f1 score of 5-fold cross validation
	Fitting	Decision trees are pruned to avoid overfitting
	Regularization	No
	Availability of configuration	Yes: https://github.com/oligogenic/VarCoPP2.0
Model	Interpretability	Transparent model, 400 decision trees
	Output	Probability, thresholded to classification
	Execution time	10000 samples in .2 seconds
	Availability of software	ORVAL: https://orval.ibsquare.be & Github: https://github.com/oligogenic/VarCoPP2.0
Evaluation	Evaluation method	Both stratified LOGO cross validation and independent validation data
	Performance measures	Average precision score, Precision, Recall, Specificity, F1 and Geometric mean
	Comparison	Confusion matrix and aforementioned performance methods on previous version of model and retrained model on new data
	Confidence	Performance differences apparent
	Availability of evaluation	Yes: Github: https://github.com/oligogenic/VarCoPP2.0

## Data Availability

VarCoPP2.0 is available through the ORVAL platform. Experiments and data through https://github.com/oligogenic/VarCoPP2.0. Version linked to manuscript at 10.5281/zenodo.6701459. *Project name* VarCoPP2.0 Project home page: https://github.com/oligogenic/VarCoPP2.0. *Operating system(s)* Platform independent. *Programming language* Python. *Other requirements* Python3.8 or higher, scikit-learn1.1.1,numpy1.21,pandas1.3 and imbalanced-learn0.9. *License* GNU GPL. *Any restrictions to use by non-academics* Written permission should be obtained.

## Abbreviations

ML: Machine Learning
VarCoPP: Variant Combination Pathogenicity Predictor
1KGP: 1000 Genomes Project
DIDA: Digenic disease database
FP: False Positive
TP: True Positive
RF: Random Forest
OLIDA: Oligogenic disease database
MAF: Minor Allele Frequency
ISPP: Inheritance Specific Pathogenicity Predictor
AD: Autosomal dominant
AR: Autosomal recessive
GO: Gene ontology
HPO: Human phenotype ontology
PPI: Protein–protein interactions
RVIS: Residual Variation Intolerance Score
LOGO: Leave-One-Group-Out
ROC: Receiver Operating Characteristic
AUC: Area Under the Curve
APS: Average Precision Score

## Footnotes

1 https://orval.ibsquare.be.

2 https://github.com/jeremymcrae/hpo_similarity.

3 http://www.github.com/andosa/treeinterpreter.

4 http://www.github.com/andosa/treeinterpreter
